# Monoclonal Antibodies Targeting Porcine Macrophages Are Able to Inhibit the Cell Entry of Macrophage-Tropic Viruses (PRRSV and ASFV)

**DOI:** 10.3390/v17020167

**Published:** 2025-01-24

**Authors:** Shaojie Han, Dayoung Oh, Nathalie Vanderheijden, Jiexiong Xie, Nadège Balmelle, Marylène Tignon, Hans J. Nauwynck

**Affiliations:** 1 Laboratory of Virology, Department of Translational Physiology, Infectiology and Public Health, Faculty of Veterinary Medicine, Ghent University, Salisburylaan 133, 9820 Merelbeke, Belgium; 2 Service Viral Re-Emerging, Enzootic and Bee Diseases, Department Infectious Diseases in Animals, Sciensano, Groeselenbergstraat 99, 1180 Brussels, Belgium

**Keywords:** porcine reproductive and respiratory syndrome virus, African swine fever virus, macrophage, monoclonal antibody, receptor, entry

## Abstract

Porcine reproductive and respiratory syndrome virus (PRRSV) and African swine fever virus (ASFV) cause serious economic losses to the swine industry worldwide. Both viruses show a tropism for macrophages, based on the use of specific entry mediators (e.g., Siglec-1 and CD163). Identifying additional mediators of viral entry is essential for advancing antiviral and vaccine development. In this context, monoclonal antibodies (mAbs) are valuable tools. This study employed a library of 166 mAbs targeting porcine alveolar macrophages (PAMs) to identify candidates capable of blocking early infection stages, including viral binding, internalization, and fusion. Immunofluorescence analysis revealed 74 mAbs with cytoplasmic staining and 70 mAbs with membrane staining. Fifteen reacted with blood monocytes as determined by flow cytometry. mAb blocking assays were performed at 4 °C and 37 °C to analyze the ability of mAbs to block PRRSV and/or ASFV infections in PAMs. The mAb 28C10 significantly blocked PRRSV (96% at 4 °C and 80% at 37 °C) and ASFV (64% at 4 °C and 81% at 37 °C) infections. The mAb 28G10B6 significantly blocked PRRSV (86% at 4 °C and 74% at 37 °C) and partially blocked ASFV (35% at 4 °C and 64% at 37 °C) infections. mAb 26B8F5-I only partially blocked PRRSV infection (65% at 4 °C and 46% at 37 °C). Western blotting and mass spectrometry identified the corresponding proteins as Siglec-1 (28C10; 250 kDa), MYH9 (28G10B6; 260 kDa), and ANXA1 (26B8F5-I; 37 kDa). Our findings are indicative that Siglec-1, MYH9, and ANXA1 play a role in PRRSV/ASFV entry into macrophages.

## 1. Introduction

The global swine industry faces significant challenges from viral diseases that result in substantial economic losses. Among the most devastating viruses are porcine reproductive and respiratory syndrome virus (PRRSV) and African swine fever virus (ASFV), both notorious for their severe impact on swine health and productivity [1,2]. PRRSV, the causative agent of porcine reproductive and respiratory syndrome (PRRS), is an enveloped single-stranded RNA virus that belongs to the genus Arterivirus, family *Arteriviridae*, order *Nidovirales* [3,4]. African swine fever (ASF), a lethal viral hemorrhagic disease of domestic pigs first reported in East Africa in 1921, is caused by ASFV, a double-strand DNA virus, the sole member of the genus *Asfivirus* within the *Asfarviridae* family [5,6]. Both PRRSV and ASFV have developed sophisticated mechanisms to enter and infect host cells. These viruses primarily target monocytes and macrophages, exploiting specific cellular receptors to gain entry. PRRSV infects host cells relying on specific cellular receptors and endocytosis to complete the viral life cycle. The interactions between viral structural proteins and cellular receptors are thought to determine the tissue tropism and host range for viruses. PRRSV facilitates its entry into host cells using receptors such as heparan sulfate, sialoadhesin (Siglec-1), Siglec-10, vimentin, MYH9, DC-SIGN, CD151, and CD163 [7,8,9,10,11]. Numerous studies have demonstrated that CD163 is essential for mediating PRRSV internalization and disassembly, determining the susceptibility of target cells to PRRSV [9,12,13]. Furthermore, additional molecules are involved in facilitating viral entry. Wei et al. found that PRRSV exposes phosphatidylserine (PS) on its envelope, mimicking apoptotic debris. The PS receptor TIM-1/4 recognizes PRRSV and induces a downstream signaling pathway that facilitates viral infection through the CD163-dependent micropinocytosis [14]. Wang et al. revealed that heat shock protein member 8 (HSPA8) interacts with PRRSV glycoprotein 4 (GP4), contributing to the virus attachment and internalization during infection [15].

Previous studies have demonstrated that ASFV can utilize both endocytosis and macropinocytosis pathways for entry into host cells [16,17]. ASFV has been shown to utilize entry mediators like CD163 and Siglec-1 to infect macrophages [18,19]. CD163-specific antibodies can reduce ASFV infection following incubation with macrophages [18,20]. However, the role of CD163 in ASFV infection is controversial, as CD163 gene knockout pigs show no resistance to ASFV infection [21]. Furthermore, several host factors such as CD45 and major histocompatibility complex class II (MHC II) are thought to play a role in ASFV attachment [20]. In addition, numerous studies have shown that ASFV will exploit apoptotic mimicry to promote its replication and spread. Gao et al. showed that ASFV induces apoptosis in PAMs at the late stage of infection, leading to the productive shedding of apoptotic bodies. These apoptotic bodies are then engulfed by neighboring PAMs, initiating secondary infections [22]. Other research demonstrated that ASFV externalized phosphatidylserine (PS) on the envelope functioned as viral apoptotic mimicry, which interacts with AXL, a tyrosine kinase receptor, to facilitate the internalization of ASFV virions via macropinocytosis entry into PAMs [23]. ASFV CD2v (the main envelope glycoprotein) interacts with CSF2RA (a hematopoietic receptor superfamily member in myeloid cells and a key receptor protein that activates receptor-associated JAK and STAT proteins) to regulate the JAK2-STAT3 pathway, inhibiting apoptosis and facilitating virus replication [24]. Despite extensive studies, the cellular and molecular mechanisms of ASFV entry into susceptible cells remain poorly understood, especially early invasion events such as receptor mediated attachment and binding.

Cell membrane receptors play a crucial role in viral binding, internalization, and genome release. A deeper understanding of the viral entry mechanisms, particularly the identification of viral receptors and ligands, is essential for novel antiviral therapies and vaccines. Previous studies in our laboratory have identified a monoclonal antibody (41D3) that is capable of blocking PRRSV infection of PAMs and identified Siglec-1 (=Sialoadhesin (Sn)) as an important target protein [25]. Thus, monoclonal antibodies against macrophages may be used to better understand cell entry mechanisms. mAbs are invaluable tools in biomedical research due to their specificity and versatility. They can be used to identify and characterize cellular proteins, elucidate their functions, and block specific biological processes. In the context of viral infections, mAbs can inhibit viral entry, neutralize viral particles, and may serve as diagnostic tools.

This study aimed to block the early stages of PRRSV/ASFV infection, including binding, internalization, and fusion, through the use of mAbs. We aimed to identify and characterize proteins on PAMs that may mediate the entry of PRRSV and ASFV.

## 2. Material and Methods

### 2.1. Cells

Primary porcine alveolar macrophages (PAMs) were obtained from 4-to-6-week-old conventional pigs from a PRRSV-negative herd, as described previously [26]. PAMs were maintained in Roswell Park Memorial Institute (RPMI 1640) medium (Gibco, Paisley, UK), supplemented with 10% fetal calf serum (FCS) (Sigma-Aldrich, St. Louis, MO, USA), 1 mM sodium pyruvate (Gibco, Grand Island, NY, USA), 1% non-essential amino acid (Gibco, Grand Island, NY, USA), 0.05 mg/mL gentamycin (Invitrogen, Gent, Belgium), 0.1 mg/mL streptomycin (Certa, Eigenbrakel, Belgium), and 100 U/mL penicillin (Kela, Sint-Niklaas, Belgium). PK15Sn^+^CD163^+^ cells were cultivated as described before [13].

### 2.2. Virus

PRRSV 13V091 (type 1 strain that was isolated in 2013 from the serum of a young piglet from a Belgian farm experiencing severe respiratory problems [27]) was used at the fifth passage in PK15Sn^+^CD163^+^ cells with a titer of 10^6.63^ TCID_50/mL_. ASFV Belgium 2018/1 (Belgian isolate, obtained in 2018 from a dead ASFV-positive wild boar in the village Etalle in Belgium [28]) was used at the sixth passage in PAMs with a titer of 10^7.47^ TCID_50/mL_.

### 2.3. Production of Monoclonal Antibodies

The library of monoclonal antibodies was previously prepared in our lab. In brief, to produce mAbs that were specifically directed against macrophages, 10-week-old female Balb/c mice were immuno-tolerized by injecting them with 1 × 10^7^ freshly isolated PBMCs and cyclophosphamide to induce immunosuppression [29]. This treatment was repeated three times biweekly. Post-treatment, mice were immunized with alveolar macrophages. Afterwards, spleen cells from the most reactive mouse were fused with SP2/0 myeloma cells. The hybridomas were screened using a cell-ELISA, and the isotype of the monoclonal antibody was identified using a monoclonal antibody isotyping kit (Bio-Rad, Lokeren, Belgium and Thermo Fisher ELISA kits, Rockford, IL, USA) following the manufacturer’s instructions.

### 2.4. The Reactivity of Monoclonal Antibodies Binding to PAMs

To determine whether the generated monoclonal antibodies were reactive to PAMs, immunofluorescence staining (IF) was performed on PAMs. In brief, PAMs were plated into 24-well plates at a concentration of 10^6^ cells per well and incubated for 24 h at 37 °C with 5% CO_2_ before IF staining. Cells were fixed and/or permeabilized using three methods before staining: (i) cell membrane staining, where cells were fixed with 4% paraformaldehyde (4% PF), and (ii) cytoplasm staining, where cells were either fixed with 4% formaldehyde and then permeabilized with 0.1% Triton or fixed and permeabilized with 100% methanol. Afterwards, a monoclonal antibody was added during a 1 h incubation at 37 °C. Then, the cells were washed with PBS and further incubated with a secondary antibody, goat-anti-mouse IgG FITC, for another 50 min at 37 °C. Following this, Hoechst staining was applied to stain the nuclei, and the cells were incubated for an additional 10 min at 37 °C. Cells were washed, and plates were read by a fluorescent microscope (Leica Microsystems GmbH, Heidelberg, Germany).

The reactivity of produced antibodies to PAMs was examined depending on the level of staining with a fluorescence microscope (Leica Microsystems GmbH, Germany): − (negative, no reactivity), + (low positive), +++ (clear positive), and +++++ (strong positive). A heatmap was plotted by https://www.bioinformatics.com.cn (last accessed on 20 May 2024), an online platform for data analysis and visualization [30].

### 2.5. Flow Cytometry

To test the reactivity of the produced monoclonal antibodies against lymphocytes and/or monocytes, PBMCs were freshly isolated as described before [31]. These cells were then stained with the produced antibodies and other known blood lymphocyte and blood monocyte markers (Table 1) and analyzed by flow cytometry. Briefly, 5 × 10^5^ isolated PBMCs were seeded into a 96-well plate. The cells were washed once with RPMI 1640 medium containing 1 mM EDTA (VWR International, Leuven, Belgium) and 1% FCS. After washing, the cells were incubated with primary antibodies for 60 min at 4 °C. Following another wash, the cells were incubated with secondary antibodies for 45 min at 4 °C. Cell viability was assessed using SYTOX™ Blue Dead Cell Stain, for flow cytometry (Thermo Fisher Scientific, Eugene, OR, USA) according to the manufacturer’s instructions. The cells were then analyzed on a Beckman CytoFLEX flow cytometer. A total of 10,000 events were recorded and displayed, and doublets were excluded with a gating strategy based on forward light scatter and side light scatter. Acquired data were analyzed by CytExpert 2.3 software (Beckman Coulter, Brea, CA, USA).

### 2.6. Virus mAb Blocking Assay

To test if anti-PAMs antibodies (hybridoma supernatants) could protect PAMs from PRRSV and/or ASFV infection, a virus mAb blocking assay was performed based on previous work [32,35], with some modifications. Briefly, PAMs were plated at 10^6^ cells/mL (1 mL/well) in a 24-well plate and incubated for 24 h at 37 °C with 5% CO_2_. Afterwards, 250 µL of the hybridoma supernatants were added per well as pre-treatment, followed by a 1 h incubation at 4 °C or 37 °C. The cells were then washed and inoculated with 125 µL of virus (PRRSV: 10^5.7^ TCID_50_/mL, ASFV: 10^6.5^ TCID_50_/mL) and 125 µL of hybridoma supernatants per well for 1 h at 4 °C (virus attachment) and 37 °C (virus attachment and internalization). After inoculation, the cells were washed and refed with 250 µL of culture medium and 250 µL of hybridoma supernatants (post-treatment). Three control groups were introduced during the virus mAb blocking assay: (i) positive controls—monoclonal antibodies against Sn (mAb 41D3; blocks binding) and CD163 (mAb 2A10; blocks disassembly) [18,32]; (ii) mock controls—mouse-anti-PCV2 capsid mAb 12E12 (IgG2b) [36] and mouse-anti-PCV2 capsid mAb 38C1 (IgG1) [37]; and (iii) negative control—triplicate of hybridoma cell culture medium (HCM) [34]. The cells were fixed with 4% PF and permeabilized with 0.1% triton 12 h (for PRRSV) or 24 h (for ASFV) post-inoculation. Viral antigen-positive cells were evaluated by immunofluorescence staining as described before [38,39]. The total number of cells and viral antigen-positive cells were quantitated from 10 randomly selected fields (10× ocular lens and a 40× objective) using a fluorescent microscope (Leica Microsystems GmbH, Heidelberg, Germany).

The blocking effect of the virus (attachment, binding, and internalization) by mAbs was calculated using the following formulas and represented as the percentage of infection relative to controls:

1. Percentage of infected cells with a certain mAb: viral antigen-positive cells/total number of cells.
2. Mean percentage of infected cells with control mAbs + HCM: (percentage of infected cells (%) with 38C1 + % with 12E12 + % with HCM1 + % with HCM2 + % with HCM3)/5.

Percentage of infected cells with a certain mAb relative to controls: percentage of infected cells incubated with a certain mAb/mean percentage of infected cells with control mAbs + HCM × 100.

### 2.7. Western Blot

To identify the reactive protein of the test mAb on PAMs by Western blot, PAM lysates were prepared in RIPA buffer (50 mM Tris-HCl, pH 8.0, 1% NP-40, 0.1% SDS, 0.5% sodium deoxycholate, 1 mM EDTA, 150 mM NaCl, 1% protease inhibitor). Samples were mixed with reducing (+ beta-mercaptoethanol) or non-reducing (- beta-mercaptoethanol) Laemmli buffer (4×) SDS-PAGE loading dye, boiled for 10 min, and subjected to SDS-PAGE (10% gel) using a Bio-Rad Mini Protean system. Proteins were transferred to a PVDF membrane (GE Healthcare) using a Bio-Rad mini trans-blot system. The membranes were blocked for 1 h at room temperature with blocking solution (5% skimmed milk in PBS, 0.1% Tween-20). The mAbs were diluted 1:50 in blocking solution and incubated with the membrane overnight at 4 °C. Peroxidase-labeled goat-anti-mouse IgG antibody (Dako, Santa Clara, CA, USA), diluted 1:2000 in blocking solution, was used for the detection of the target protein–mAb complex. The results were visualized with an ECL Western blotting detection system (GE Healthcare).

### 2.8. Deglycosylation Assay

To determine whether the target protein of a certain mAb was glycosylated, PAM lysates were either left untreated or treated with PNGase F (New England Biolabs Inc., Ipswich, MA, USA) for 3 h at 37 °C following the manufacturer’s instructions. Afterwards, samples were mixed with reducing Laemmli buffer (4×) SDS-PAGE loading dye, boiled for 10 min, and subjected to SDS-PAGE and Western blot analysis.

### 2.9. Immunoprecipitation

To further characterize the mAb-interacting protein in PAMs, immunoprecipitation was performed. Briefly, 500 µL of mAb (hybridoma supernatant) was incubated with 50 µL of Protein A/G magnetic beads or Protein L magnetic beads (Thermo Fisher, Rockford, IL, USA) at room temperature for 1 h while shaking. After incubation, unbound mAbs were removed using a magnetic stand, and 500 µL of PAM lysate (50mM Tris HCl, pH 8.0; 150 mM NaCl; 1% NP-40; 0.5% sodium deoxycholate and 0.1% SDS) was added and incubated with the Protein A/G magnetic beads or Protein L magnetic beads bound with mAb at 4 °C overnight while shaking. The proteins bound to the beads were eluted with SDS-PAGE loading buffer under reducing and non-reducing conditions. Subsequently, the proteins were subjected to SDS-PAGE (10% gel) using a Bio-Rad Mini Protean system. One gel was stained with Coomassie blue dye (Bio-Rad, Lokeren, Belgium) after running, while the proteins in another gel were transferred to a PVDF membrane using a Bio-Rad mini trans-blot system. The membranes were blocked for 1 h at room temperature with blocking solution (5% skimmed milk in PBS, 0.1% Tween-20). The test mAbs were diluted 1:50 in blocking solution and incubated with the membrane overnight at 4 °C. Peroxidase-labeled goat-anti-mouse IgG antibody (Dako), diluted 1:2000 in blocking solution, was used to detect the target protein of the test mAb. The results were visualized using an ECL Western blotting detection system (GE Healthcare, Buckinghamshire, UK). The mAb target protein were cut from the gel and sent for mass spectrometry analysis.

### 2.10. Mass Spectrometry

Liquid Chromatography with tandem mass spectrometry (LC-MS-MS) runs of gel samples were searched separately using the MaxQuant algorithm (version 2.4.13.0) with mainly default search settings, including a false discovery rate set at 1% on PSM and protein level. Spectra were searched against the following protein sequence database: Sus scrofa (taxid 9823) Uniprot reference database (www.uniprot.org, accessed on 30 October 2024). The most abundant protein hits were identified by using the info iBAQ (intensity value) and relative abundance within the gel band. IBAQ intensity is calculated as follows: sum of the peptide intensities/# detectable tryptic peptides.

### 2.11. Statistics Analysis

Statistical analysis was performed using GraphPad Prism version 9.0. Statistical significance between the two groups was analyzed using unpaired Student’s *t*-test, and differences between three or more groups were compared with a control group using a one-way analysis of variance (ANOVA).

*p* < 0.05 was considered to indicate a statistically significant difference.

## 3. Results

### 3.1. Characterization of Monoclonal Antibodies Against PAMs

**Figure 1:**
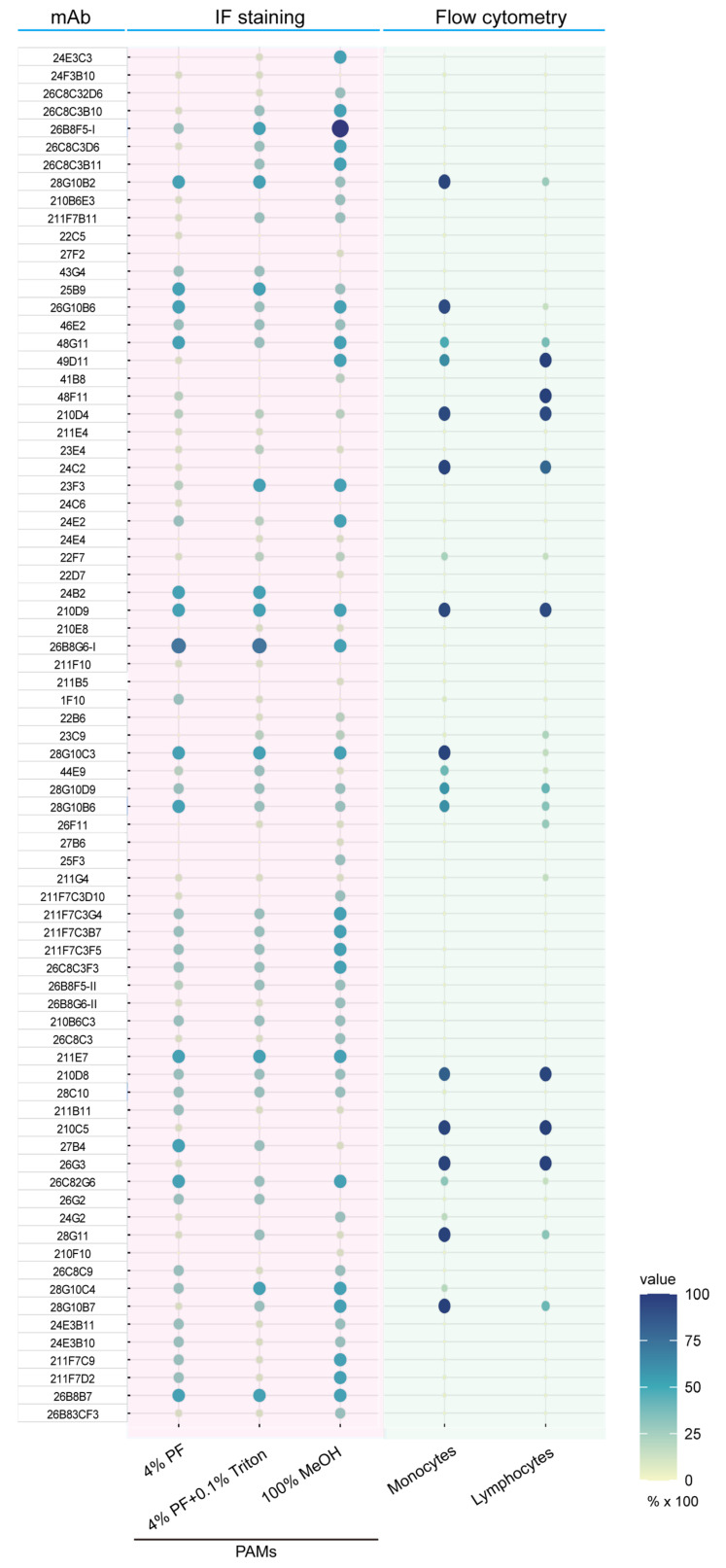
The reactivity of mAbs to PAMs was evaluated by IF staining and flow cytometry. The PAMs were fixed and permeabilized using three different methods before staining: (i) cell membrane staining with 4% PF and (ii) cytoplasm staining with 4% formaldehyde followed by permeabilization with 0.1% Triton (4% PF+ 0.1% Triton) or 100% methanol (100% MeOH). The reactivity of the produced antibodies to the PAMs was examined using a fluorescence microscope (Leica Microsystems GmbH, Heidelberg, Germany) and scored as negative, low positive, clear positive, or strong positive. The reactivity against monocytes (SWC3^+^ cells) and/or lymphocytes (CD3^+^ cells) was evaluated by flow cytometry. The size and color of the bubbles correlate with the reactivity of mAbs to the cells under different conditions.

**Figure 2:**
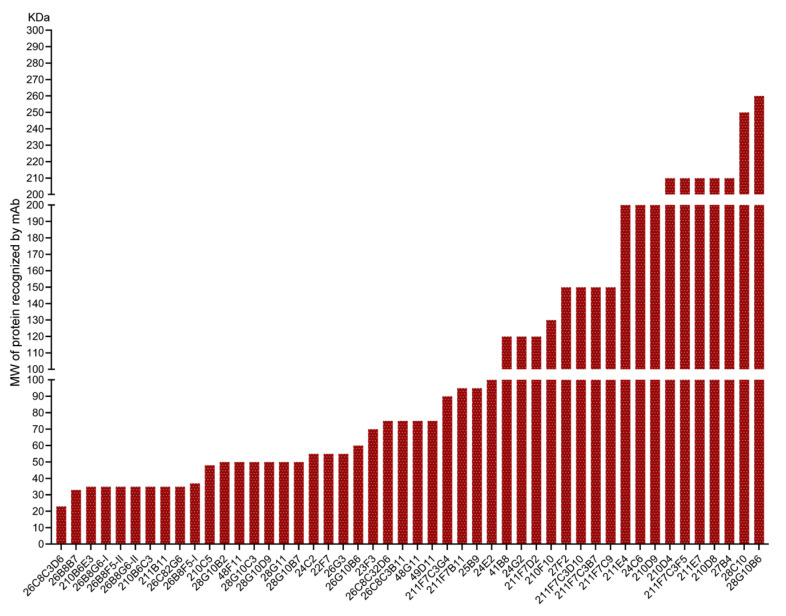
Evaluation of the apparent molecular weight of the target proteins of mAbs in PAMs using Western blotting. Porcine alveolar macrophages (PAMs) were lysed using a RIPA buffer under reducing and non-reducing conditions. Subsequently, the proteins from the lysates were separated using SDS-PAGE. Different monoclonal antibodies (mAbs) were then used for immunoblotting the proteins from the lysates.

Initially, a total of 166 mAb-secreting hybridoma cell lines were produced, and 77 of these mAbs showed reactivity to PAMs after an IF staining (Figure 1). A total of 74 mAbs showed a cytoplasmic staining in permeabilized cells, and 70 mAbs showed a membrane staining in non-permeabilized cells. Among these, forty-eight mAbs were isotyped as IgG1, three as IgG2b, one as IgG3, and twenty-three as IgM. Next, to determine the specificity of the mAbs against macrophages, the reactivity of the mAbs with monocytes and lymphocytes was tested using flow cytometry with different cell markers: SWC3 for monocytes and CD3 for lymphocytes. Twenty of these mAbs reacted against blood monocytes and twenty-two mAbs against lymphocytes. Next, a Western blot was performed to identify the target protein of mAbs in PAM lysate. A protein band was observed in 48 out of the 77 mAbs. The apparent molecular weight of the target proteins ranged from 23 kDa to 300 kDa (Figure 2). These data demonstrate that many of our newly produced mAbs showed a strong reactivity with macrophages.

### 3.2. Screening of mAbs That Block PRRSV/ASFV Entry

**Figure 3:**
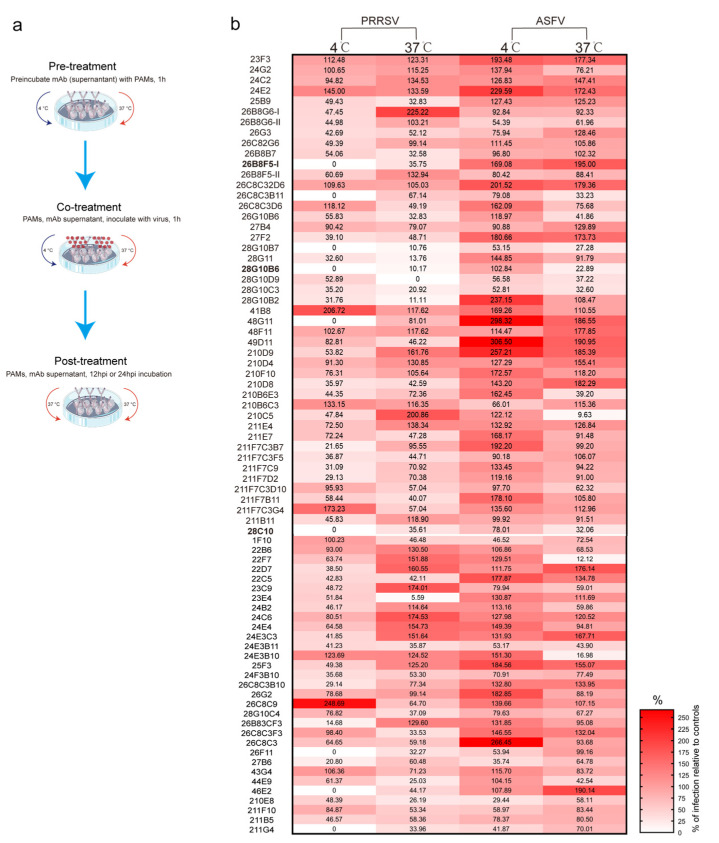
The screening of mAbs using virus mAb blocking assay. (**a**) Scheme of process of virus mAb blocking assay. PAMs were plated at 10^6^ cells/mL (1 mL/well) in a 24-well plate and incubated for 24 h at 37 °C. Afterwards, 250 µL hybridoma supernatants were added per well, followed by a 1 h incubation at 4 °C or 37 °C. The cells were then washed and inoculated with 125 µL virus (PRRSV: 10^5.7^ TCID_50_/mL, ASFV: 10^6.5^ TCID_50_/mL) and 125 µL of hybridoma supernatant per well at the same temperature. After inoculation, the cells were washed and refed with 250 µL of culture medium and 250 µL of hybridoma supernatant. (**b**) Heatmap representing the percentage of infection relative to controls of 77 mAbs after PRRSV/ASFV mAb blocking analysis. The mAbs were clustered based on their clone number.

To assess whether mAbs can block viral entry during the viral replication cycle, viral mAb blocking assays were conducted as previously described [32], with some modifications. Cells were incubated with renewed addition of mAbs at three different timepoints: before (pre-treatment), during (co-treatment), and after (post-treatment) viral inoculation (Figure 3a). A specific anti-Sn monoclonal antibody (41D3) and a specific anti-CD163 monoclonal antibody (2A10), previously reported to be able to block PRRSV and ASFV infection in PAMs [18,25,32], were included as a positive control in the PRRSV/ASFV mAb blocking assay (see Figure S1). In the first round of preliminary selection, 77 mAbs which showed a reactivity with PAMs were introduced into the virus mAb blocking assay (Figure 3b). Eleven mAbs showed a blocking effect on PRRSV infection (percentage of relative infection at 4 °C: 0 to 63%; percentage of relative infection at 37 °C: 6% to 80%), fifteen mAbs showed a blocking effect on ASFV despite a fainter inhibition compared to PRRSV (percentage of relative infection at 4 °C: 29% to 100%; percentage of relative infection at 37 °C: 10% to 72%), and six mAbs showed blocking effects on both PRRSV and ASFV (Figure S1). Fetal calf serum (FCS) is commonly included in the initial cell growth medium to support cell growth; however, it can inhibit PRRSV infection due to the presence of protease inhibitors [40]. To exclude FCS’s impact on the blocking assay results, FCS-free conditions were introduced in the new mAb production process. The newly produced mAbs (-FCS) were initially analyzed for their reactivity on PAMs by IF staining, and their concentrations were measured (Table S1). Subsequently, the virus blocking assay was performed using the newly produced mAbs for second-round screening (Figure S2).

**Figure 4:**
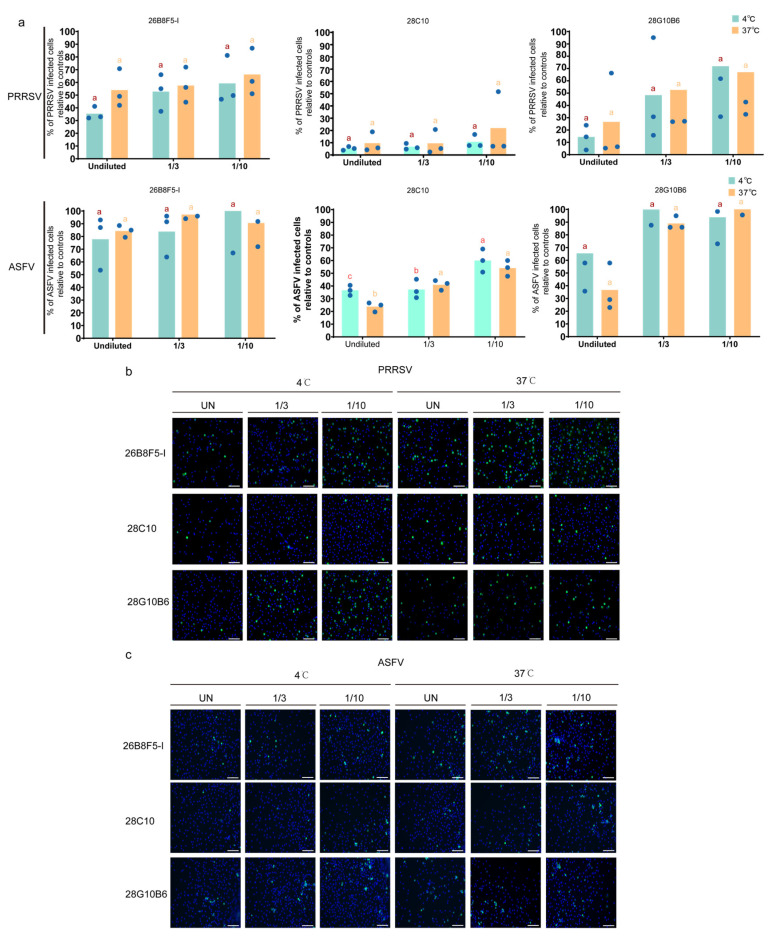
Validation of three selected mAbs for virus blocking effect. (**a**) The top 3 selected mAbs (26B8F5-I, 28C10, and 28G10B6) were produced in culture medium without FCS, and PRRSV/ASFV blocking assays were performed with different dilutions (undiluted (UN), 1/3 diluted (1/3), and 1/10 diluted (1/10). Statistical significance was determined by one-way ANOVA followed by Tukey’s multiple comparison post hoc test. Different letters represent significant differences (*p* < 0.05). The results are presented as the mean ± standard deviation of three independent measurements. (**b**,**c**) The viral antigen-positive cells (green fluorescence) were visualized by immunofluorescence staining after mAb virus blocking assay. Nuclei were counterstained in blue (Hoechst). Scale bar: 100 µm.

Based on the reactivity of mAbs with PAMs and their blocking effect on PRRSV or ASFV, the top three mAbs (26B8F5-I, 28C10, and 28G10B6) were selected to repeat the virus mAb blocking assay at different concentrations (Figure 4).

For PRRSV, 26B8F5-I showed a clear blocking effect, with 65% blocking at 4 °C and 46% blocking at 37 °C. Similarly, 28C10 exhibited a strong blocking effect, achieving 96% blocking at 4 °C and 80% blocking at 37 °C. 28G10B6 also demonstrated strong blocking effects, with 86% blocking at 4 °C and 74% blocking at 37 °C.

For ASFV, 26B8F5-I displayed only a slight blocking effect, with 22% blocking at 4 °C and 16% blocking at 37 °C. In contrast, 28C10 showed a moderate blocking effect, achieving 64% blocking at 4 °C and 81% blocking at 37 °C. 28G10B6 exhibited moderate blocking effects as well, with 35% blocking at 4 °C and 64% blocking at 37 °C.

These results demonstrate that our selected mAbs effectively block viral entry into PAMs, suggesting that the target proteins of these mAbs are involved in mediating early viral entry into PAMs.

### 3.3. Identification of Target Proteins of the Three Selected mAbs by LC-MS/MS

Next, to determine the identities of the proteins recognized by these mAbs, immunoprecipitation with corresponding mAbs was performed, and the proteins of interest were subsequently analyzed by mass spectrometry.

**26B8F5-I**

**Figure 5:**
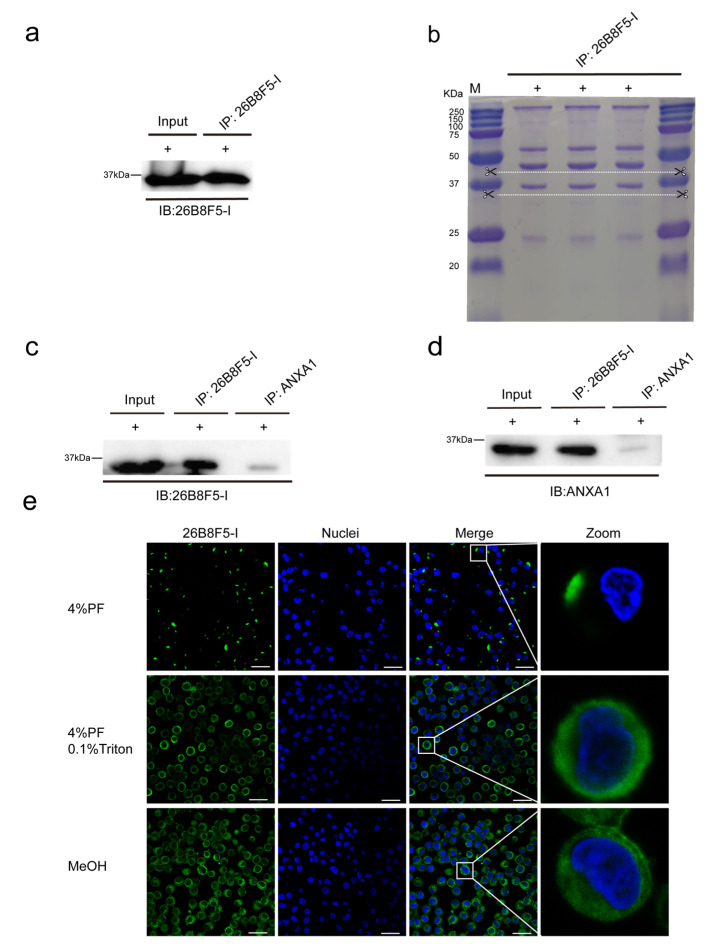
Identification and characterization of the target protein reacting with mAb 26B8F5-I. (**a**–**d**) Western blotting and immunoprecipitation were used to detect and purify the proteins that react with mAb 26B8F5-I. The PAM lysates were prepared in the RIPA buffer under reducing condition (+). The lysates were incubated with protein A/G magnetic beads which were preincubated with mAb 26B8F5-I, and the proteins that reacted with the mAb were eluted from protein A/G magnetic beads and analyzed on SDS-PAGE, followed by Western blotting. The whole PAM lysates (Input) and immunoprecipitates were immunoblotted with mAb 26B8F5-I and pAb against ANXA1, respectively. (**e**) Immunofluorescence staining was performed to determine the subcellular localization of the protein in response to mAb 26B8F5-I under various fixation conditions: (1) 4% PF: cells were fixed with 4% paraformaldehyde, (2) 4% PF and 0.1% Triton: cells were fixed with 4% paraformaldehyde, followed by permeabilization with 0.1% Triton, and (3) 100% MeOH: cells were fixed with 100% methanol. Nuclei were counterstained in blue (Hoechst). Scale bar: 25 µm.

Briefly, a 37 kDa protein in the PAM cell lysate was recognized by mAb 26B8F5-I in Western blot, and the same apparent molecular weight protein was revealed again after immunoprecipitation (Figure 5a). Following separation by SDS-PAGE and staining with Coomassie blue, the protein band near 37 kDa (between the two white dashed lines) was excised from the gel for mass spectrometry identification (Figure 5b). As shown in Table S2, Annexin A1 (ANXA1) was identified as the most abundant protein in the sample after immunoprecipitation with mAb 26B8F5-I. To confirm that ANXA1 is the real target protein of our mAb 26B8F5-I, a commercial polyclonal antibody (pAb) against ANXA1 was used in Western blot and immunoprecipitation analyses. As shown in Figure 5c,d, the pAb against ANXA1 demonstrated reactivity with the PAMs lysate and the immunoprecipitate of mAb 26B8F5-I, while our mAb 26B8F5-I specifically recognized the immunoprecipitate of ANXA1, revealing a 37 kDa protein and confirming Annexin A1 as the target protein of mAb 26B8F5-I. Furthermore, using our mAb (26B8F5-I) for immunofluorescence staining revealed an intriguing distribution pattern of ANXA1 in PAMs. On the cell membrane (membrane staining), ANXA1 appears as discrete patches, whereas, in the cytoplasm (after permeabilization), it is diffusely distributed (Figure 5e).

**28C10**

**Figure 6:**
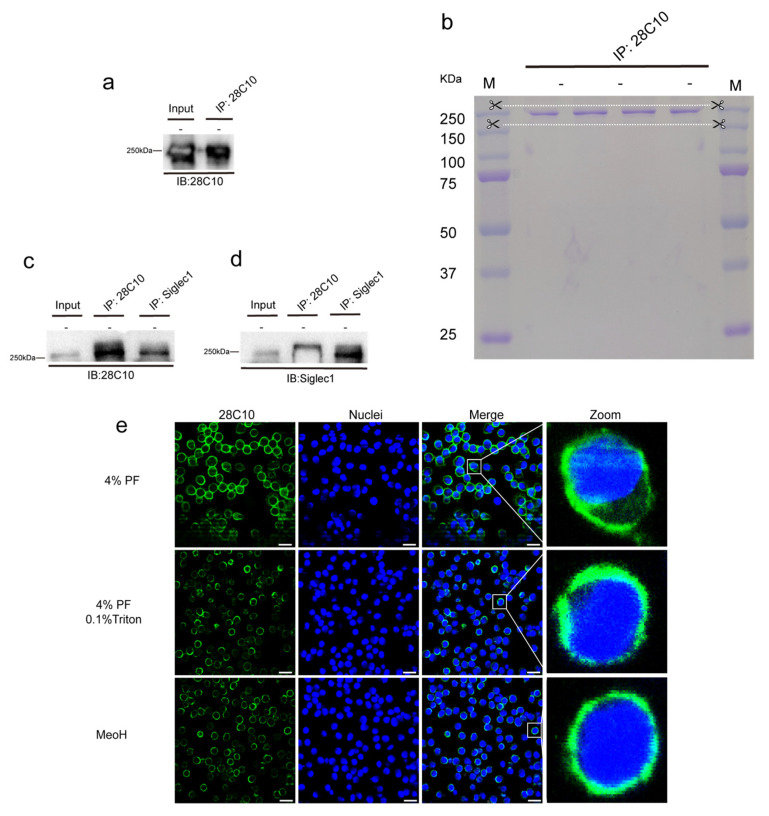
Identification and characterization of the target protein reacting with mAb 28C10. (**a**–**d**) Western blotting and immunoprecipitation were used to detect and isolate the proteins reactive with mAb 28C10. The PAM lysates were prepared in the RIPA buffer under non-reducing conditions (-). The lysates were incubated with protein A/G magnetic beads which were preincubated with mAb 28C10, and the proteins reactive with the mAb were eluted from protein A/G magnetic beads and analyzed on SDS-PAGE, followed by Western blot. The whole PAM lysates (Input) and immunoprecipitates were immunoblotted with mAb 28C10 and mAb 41D3 against Siglec-1, respectively. (**e**) Immunofluorescence staining was performed to determine the subcellular location of the protein that reacted with mAb 28C10 under different conditions: (1) 4% PF: cells were fixed with 4% paraformaldehyde, (2) 4% PF and 0.1% Triton: cells were fixed with 4% paraformaldehyde, followed by permeabilization with 0.1% Triton, and (3) 100% MeOH: cells were fixed with 100% methanol. Nuclei were counterstained in blue (Hoechst). Scale bar: 25 µm.

**Figure 7:**
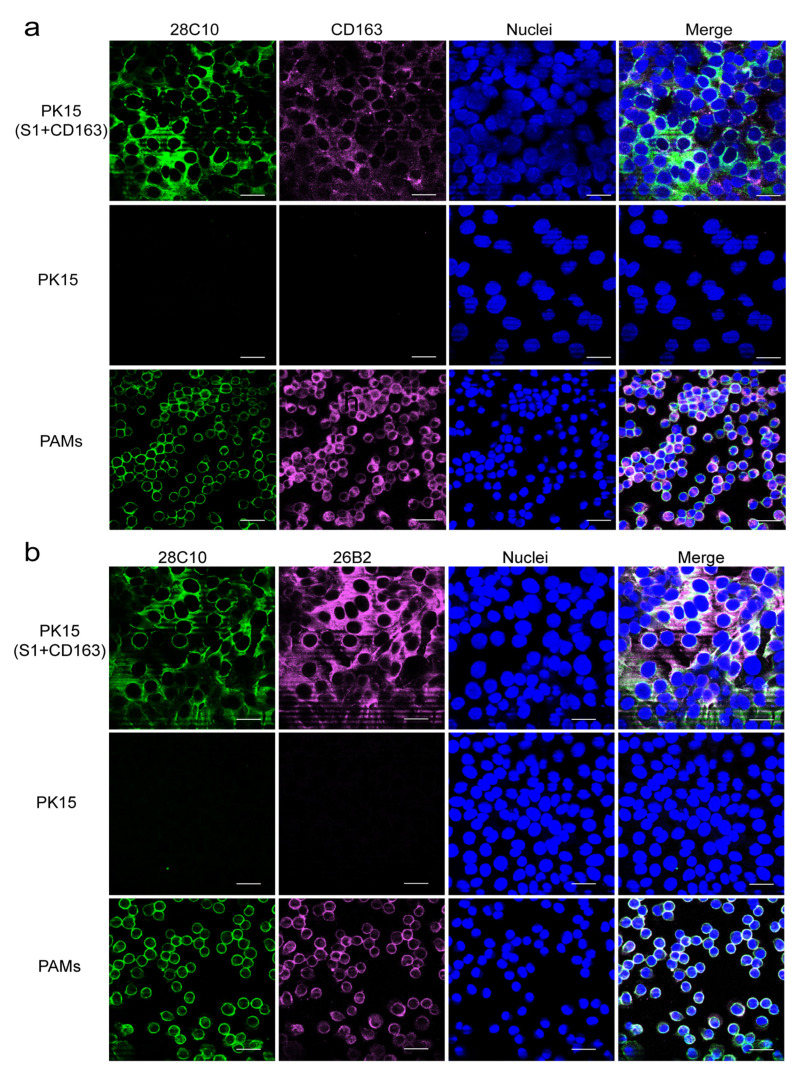
Double immunofluorescence staining against 28C10 (Siglec1) and CD163 was performed on PK15, PK15^S1-CD163^ cells, and PAMs. (**a**) Double immunofluorescence staining for 28C10 (Siglec1) (green) and CD163 (magenta) was performed on various cell types. (**b**) Double immunofluorescence staining for Siglec1 was conducted using two different mAbs (28C10 and 26B2) across different cell types. Nuclei were counterstained in blue (Hoechst). Scale bar: 25 µm.

Similarly, as shown in Figure 6a, a 250 kDa protein in the PAM lysate was recognized by mAb 28C10 under non-reducing conditions, and this protein was confirmed through immunoprecipitation. The protein band near 250 kDa (between the two white dashed lines) was excised from the gel for proteomics identification (Figure 6b). As shown in Table S3, Siglec-1 (S1, also known as Sialoadhesin (Sn)) was identified as the most abundant protein in the sample after immunoprecipitation with mAb 28C10. Another mAb against Sn (41D3), developed in our lab, also revealed a band around 250 kDa under non-reducing conditions [25]. As shown in Figure 6c,d, mAb 41D3 against Siglec-1 demonstrated reactivity with the PAM lysate and the immunoprecipitate of mAb 28C10, while our mAb 28C10 specifically recognized the immunoprecipitate of mAb 41D3, revealing a 250 kDa protein and confirming Siglec-1 as the target protein of mAb 28C10. Additionally, the protein recognized by mAb 28C10 was primarily present on the PAM membrane, as demonstrated by immunofluorescence staining under various treatments (Figure 6e). Furthermore, another mAb against human Siglec-1 (26B2, IgG2b) was introduced alongside mAb 28C10 for double IF staining on PK15^S1+CD163+^ cells. As shown in Figure 7a, Siglec-1 and CD163 were highly expressed on PK15^S1-CD163^ cells and PAMs. Moreover, colocalization of mAb 26B2 (magenta) and mAb 28C10 (green) was observed in PK15^S1-CD163^ cells and PAMs (Figure 7b).

**28G10B6**

**Figure 8:**
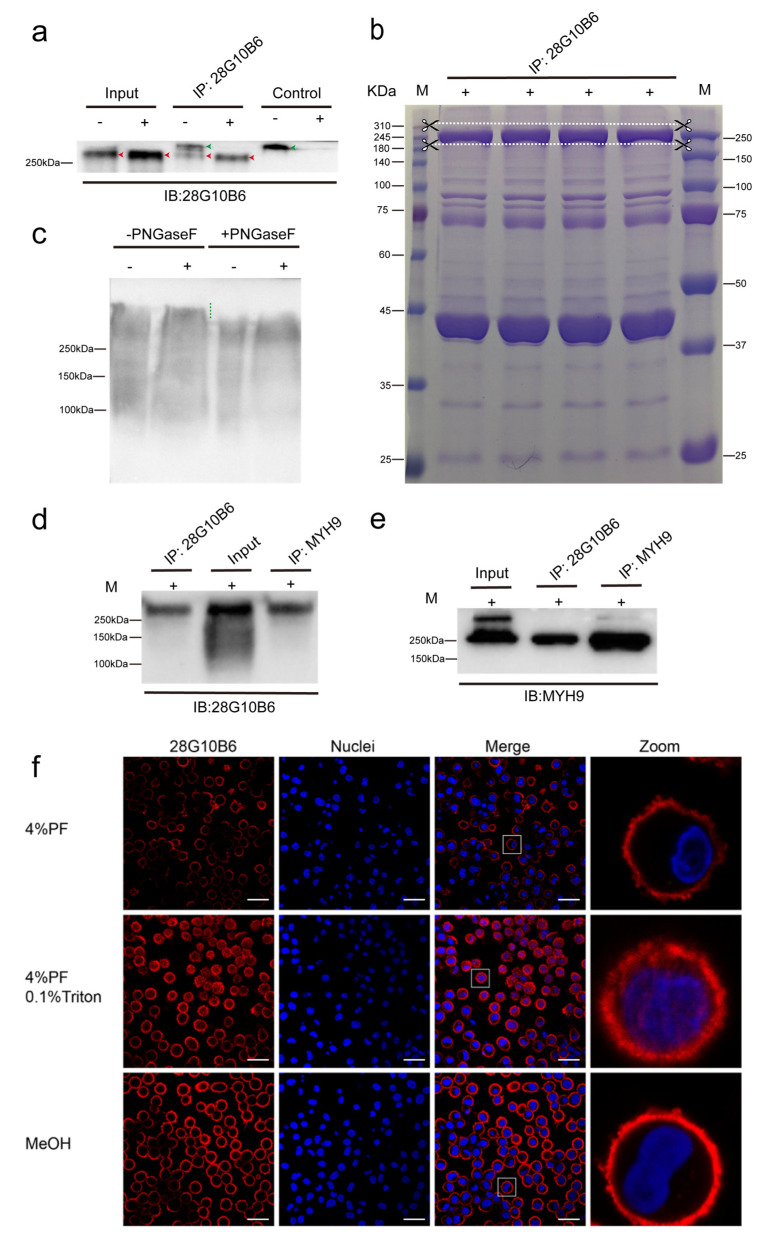
Identification and characterization of the target protein reacting with mAb 28G10B6. (**a**–**e**) Western blotting and immunoprecipitation were used to detect and isolate the proteins that react with mAb 28G10B6. The PAM lysates were prepared in the RIPA buffer under reducing (+) and non-reducing (−) conditions. The lysates were incubated with protein L magnetic beads which are preincubated with mAb 28G10B6, and the proteins that react with mAb were eluted from protein L magnetic beads and analyzed on SDS-PAGE, followed by Western blotting. For the control, RIPA buffer was incubated with Protein L beads pre-incubated with mAb 28G10B6, and the bound mAb was then eluted from the Protein L beads and analyzed by Western blotting. The whole PAM lysates were pretreated with PNGaseF or not, and then the samples were analyzed on SDS-PAGE, followed by Western blot. (**f**) Immunofluorescence staining was performed to determine the subcellular location of protein reactive with mAb 28G10B6 under different conditions: (1) 4% PF: cells were fixed with 4% paraformaldehyde, (2) 4% PF and 0.1% Triton: cells were fixed with 4% paraformaldehyde, followed by permeabilization with 0.1% Triton, and (3) 100% MeOH: cells were fixed with 100% methanol. Nuclei were counterstained in blue (Hoechst). Scale bar: 25 µm.

As shown in Figure 8a, a 260 kDa protein in the PAM cell lysate was recognized by mAb 28G10B6 under both reducing and non-reducing conditions, and this recognition was further validated through immunoprecipitation. Considering the relatively large molecular weight of the antibody (IgM, 900 kDa) compared to the target protein, a set of controls was included during the immunoprecipitation process to distinguish between the eluted capture antibody (indicated by a green arrowhead) and the target protein (indicated by a red arrowhead). The protein band near 260 kDa (indicated between the two white dashed lines) was excised from the gel for proteomic analysis (Figure 8b). Treatment with PNGaseF led to a decrease in the molecular weight of the protein recognized by mAb 28G10B6 (Figure 8c), indicating the presence of N-linked glycans. After immunoprecipitation with mAb 28G10B6, myosin heavy chain 9 (MYH9) was identified as the most abundant protein in the sample using mass spectrometry analysis, as shown in Table S4. To confirm MYH9 as the target of mAb 28G10B6, Western blot and immunoprecipitation analyses were performed using a commercial monoclonal antibody against MYH9. Figure 8d,e demonstrate that this antibody (MYH9 monoclonal antibody, 5D9D2) reacts with PAM lysate and the immunoprecipitate of mAb 28G10B6, while our mAb 28G10B6 specifically recognized the immunoprecipitate of MYH9, confirming our findings regarding the 260 kDa protein. MYH9 is a glycoprotein (ref.). Additionally, immunofluorescence staining with mAb 28G10B6 revealed a distinctive distribution pattern of MYH9 in PAMs (Figure 8f), showing widespread localization on both the cell membrane and in the cytoplasm.

## 4. Discussion

Porcine reproductive and respiratory syndrome virus (PRRSV) and African swine fever virus (ASFV) present significant challenges to the global pig industry. Vaccination remains the primary strategy for preventing viral infections, but vaccines against PRRSV show a limited efficacy, and developing a vaccine for ASFV appears even more complex and challenging. In addition, current vaccines for PRRSV or ASFV suffer from issues such as low biosafety and inadequate cross-protection against heterologous strains [41,42]. Viral entry in their target cells (mainly macrophages) depends on a highly coordinated process involving specific ligands and cellular receptors. Consequently, research is essential to elucidate the mechanism of viral invasion in host cells, which will ultimately aid in the development of innovative antiviral strategies against PRRS and ASF. In the present study, we developed and characterized three murine monoclonal hybridoma cell lines (26B8F5-I, 28C10, and 28G10B6) that stably secrete mAbs against the target proteins ANXA1, Siglec-1, and MYH9 which are expressed in the cell membrane of PAMs and reduce infection of PAMs by PRRSV and, for some of them, ASFV, suggesting that the recognized proteins play a role in the viral entry process.

Firstly, a total of 166 hybridoma cell lines were produced, out of which 77 mAbs showed reactivity to PAMs, as demonstrated by immunofluorescence staining. The subcellular localization of the target proteins recognized by the mAbs was analyzed using various fixation methods combined with immunofluorescence staining. Particularly, a significant proportion of these mAbs demonstrated a strong membrane staining reactivity (Figure 1), suggesting that the mAb-identified targeted proteins are present on the outer cell membrane. The subclass distribution of these mAbs showed that forty-eight mAbs were of IgG1 subclass, three mAbs were of IgG2b subclass, one mAb was of IgG3 subclass, and twenty-three mAbs were of IgM subclass. Additionally, all antibodies contained kappa light chains. The specificity of these mAbs was further evaluated by flow cytometry, revealing that 20 mAbs reacted to blood monocytes with varying degrees, and 22 mAbs reacted to lymphocytes with varying degrees (Figure 1). A few mAbs showed cross-reactivity between PAMs, monocytes, and lymphocytes, indicating that the target protein was broadly present in immunological cells. Western blot analysis successfully identified target proteins for 48 of the 77 mAbs, with molecular weights ranging from 23 kDa to 300 kDa. This demonstrates the broad spectrum of antigens recognized by these mAbs (see Figure 2). The 22 mAbs that failed to detect any bands in the PAM lysate may have been unable to bind their targets due to the disruption of structural epitopes by SDS treatment.

Secondly, the ability of the mAbs to block PRRSV and/or ASFV infection in PAMs was assessed. Virus mAb blocking assays were conducted at different times relative to viral inoculation (pre-treatment, co-treatment, and post-treatment), as previously reported [32,35]. These assays incorporated a positive control using previously reported anti-Sn mAb known to block PRRSV or anti-CD163 known to block ASFV infection in PAMs (Figure S1). After the initial screening, 11 of the 77 mAbs demonstrated blocking effects against PRRSV (percentage of relative infection at 4 °C: 0 to 52%; percentage of relative infection at 37 °C: 6% to 80%), and 15 mAbs showed blocking effects against ASFV (percentage of relative infection at 4 °C: 29% to 100%; percentage of relative infection at 37 °C:10% to 72%). Notably, six mAb pairs were able to block both PRRSV and ASFV, suggesting a shared host factor between these two viruses (see Figure S1). This also highlights the potential of the target protein as a promising target for antiviral drug development.

Previous research has shown that serum treatment of macrophages inhibits PRRSV infection [40]. Furthermore, due to its compositional complexity within the cell culture supernatant, the high immunoglobulin and protein content of the FCS will make it more challenging to purify monoclonal antibodies from the supernatants of hybridoma cells [43]. To address these issues, we generated mAbs without FCS (-FCS). The newly produced mAbs still exhibited strong reactivity with PAMs, as assessed by immunofluorescence staining (see Table S1), and demonstrated varying degrees of blocking effects against PRRSV and ASFV (see Figure S2).

Considering the reactivity of monoclonal antibodies with PAM in staining and Western blot analysis, as well as their virus-blocking effects, we have selected three blocking monoclonal antibodies (26B8F5-I, 28C10, and 28G10B6) for further detailed analysis. To ensure the accuracy of the results, we repeated the virus mAb blocking assays with the three selected monoclonal antibodies, using various concentration groups (Figure 4a). None of the isotype-matched control antibodies prevented virus binding, which confirms the specificity of the blocking effect of 26B8F5-I, 28C10, and 28G10B6. The mAb 26B8F5-I (IgG1), which targets macrophages (Figure 5e), demonstrated a good blocking effect against PRRSV, with 65% blocking at 4 °C and 46% blocking at 37 °C. However, it showed less pronounced effects on ASFV, with 22% blocking at 4 °C and 16% blocking at 37 °C. These findings indicate that the target protein of 26B8F5-I is involved in virus entry, particularly for PRRSV, although it is not the sole determining factor. In contrast, the mAb 28C10 (IgG1) also targeting macrophages (Figure 6e) exhibited a strong blocking effect against both PRRSV (86% at 4 °C, 74% at 37 °C) and ASFV (64% at 4 °C, 81% at 37 °C). These results indicate that the target protein of mAb 28C10 is most likely involved in the entry of both PRRSV and ASFV into PAMs. The mAb 28G10B6 (IgM), which targets macrophages (Figure 8f), monocytes, and lymphocytes, showed significant blocking effects on PRRSV (86% at 4 °C, 74% at 37 °C) and ASFV (35% at 4 °C, 63% at 37 °C), suggesting that its target protein is important for virus entry.

In our study, after excluding influencing factors such as cell status and presence of FCS, we did not observe any mAb capable of completely blocking the infection by PRRSV or ASFV in PAM. This suggests that the interaction between these viruses and macrophages is complex and likely involves multiple mediators. Several molecules have been described as entry mediators (receptor or co-receptor) for PRRSV: heparan sulphate, Siglec-1, Siglec-10, MYH9, Vimentin, CD151, DC-SIGN (CD209), CD163, Heat shock protein 8 (HSPA8), GAPDH, and TIM4 [9,10,13,14,15,44,45,46,47,48,49]. Receptors for ASFV are still a matter of debate. Several macrophage receptors have been proposed, such as CD163, Siglec-1, CD45, and MHC II. They all may play a role in the virus interaction with host cells [18,19,20].

To gain a deeper understanding of virus–cell interactions and identify the specific proteins involved in PRRSV and ASFV entry, we conducted mass spectrometry analysis on the proteins targeted by mAbs capable of blocking viral invasion. In this study, we identified A1 (ANXA1) as the target protein recognized by the mAb 26B8F5-I in PAMs (see Table S2). This discovery was based on the initial recognition of a 37 kDa protein in PAM lysates by mAb 26B8F5-I, which was consistently observed following immunoprecipitation and Western blot analysis (Figure 5a). A1 (ANXA1) is a phospholipid binding, calcium-dependent protein known to play essential roles in multiple cellular functions including inflammation, proliferation, migration, and apoptosis [50,51,52]. Previous research has shown that ANXA1 is primarily located in the cytoplasm and can be secreted to bind with negatively charged phospholipids or proteins on the cell surface through calcium interactions [53]. This observation is consistent with our findings that ANXA1 is mainly present in the cytoplasm and is distributed in patchy patterns on the cell membrane (Figure 5e). Notably, ANXA1 is a membrane-associated protein with various intracellular functions, and it is directly or indirectly involved in the replication of many viruses. ANXA1 was reported to promote influenza A virus (IAV) entry and enhance apoptosis after IAV infection [50,54]. Foot-and-mouth disease virus (FMDV) antagonizes the effect of ANXA1-promoted IFN-β production and positively modulates viral replication [55].

Several proteins were co-identified in the eluate of mAb 26B8F5-I (Table S2), indicating likely interactions with ANXA1. Such protein is A2 (ANXA2),) another member of the family of proteins. It shares a highly similar functional domain with ANXA1, as it is also known for its ability to bind phospholipids in a calcium-dependent manner. ANXA2 is expressed on macrophages and upregulated in PAMs infected with PRRSV. Together with vimentin, it interacts with the N protein of PRRSV and enhances PRRSV replication [56]. ANXA2 has been reported to play a role in the production of infectious particles of classical swine fever virus and has been identified as a novel host factor contributing to the formation of infectious hepatitis C virus (HCV) particles [57,58]. Pseudorabies virus (PRV) infection leads to the extracellular translocation of ANXA2, and inhibitors targeting ANXA2 have been shown to inhibit PRV propagation [59]. Very importantly, in addition to ANXA2, other proteins that possibly interact with ANXA1 were identified, including HSPA8, GAPDH, and vimentin. HSAP8 is involved in the attachment and internalization of PRRSV [15], while GAPDH, restricted to the cytoplasm by viral GP5, facilitates PRRSV replication through its glycolytic activity [48]. The lactate-lactylation-HSPA6 axis promotes PRRSV replication by impairing IFN-β production [60]. Previous research observed an upregulation of ANXA1 in PRRSV-infected PAMs analyzed by mass spectrometry [61]. Extensive studies of ANXA1 and its interacting proteins in the context of PRRSV infection have indirectly confirmed ANXA1’s role in mediating PRRSV entry. Future studies will utilize siRNA technology to inhibit or knock down ANXA1 expression in PAMs, with the aim of further clarifying ANXA1’s role in PRRSV invasion. Additionally, co-IP will be employed to identify key viral proteins that interact with ANXA1, helping to elucidate the molecular mechanisms of ANXA1-mediated PRRSV invasion.

Many recent studies have shown that viruses can exploit apoptotic mimicry on the virion surface to mediate viral dissemination. This mimicry is characterized by the exposure of phosphatidylserine (PS), a hallmark of apoptosis, which acts as an “eat me” signal and masquerades as apoptotic debris. This strategy is increasingly recognized as a common mechanism used by enveloped viruses to facilitate viral entry [62]. PRRSV infection triggers autophagy via endoplasmic reticulum (ER) stress-induced calcium signaling to facilitate virus replication [63]. Previous research demonstrated that PRRSV has phosphatidylserine (PS) on its envelope as a form of viral apoptotic mimicry and infects host cells via an alternative pathway involving T-cell immunoglobulin and mucin domain (TIM) receptors and CD163-mediated macropinocytosis [14]. Chen et al. demonstrated that ASFV also possess phosphatidylserine (PS) on its envelope which acts as viral apoptotic mimicry, interacting with AXL, a tyrosine kinase receptor, to mediate the internalization of ASFV into PAMs [23]. ANXA1 upregulates neutrophil apoptosis and aids in the binding of PS to phagocytic receptors. Besides initiating apoptosis, ANXA1 also plays a crucial role in the removal of apoptotic cells. It is recruited to the surface of the cells in a caspase- and calcium-dependent manner, where it co-localizes with PS [64]. Further exploration will determine whether ANXA1 interacts with PS on virions such as PRRSV and ASFV and whether it promotes viral entry and cell-to-cell spread through apoptotic mimicry.

Next, Siglec-1 (S1) was proposed by our proteomic analysis as the target protein of mAb 28C10 in PAMs. This identification was corroborated by another mAb, 41D3, which recognizes a 250 kDa protein (Figure 6c,d). The antibodies 28C10 and 41D3 may recognize distinct isoforms or post-translationally modified forms of Siglec-1, potentially accounting for the observed differences in molecular weights on the gel compared to previous findings [25]. The immunofluorescence (IF) staining analysis further confirmed the localization of Siglec-1 primarily on the PAM membrane (Figure 6e). The identification of Siglec-1 as the target protein for mAb 28C10 underscores its significant role in PRRSV and ASFV infections. Siglec-1 is known for its involvement in sialic acid-mediated cellular interactions, which are essential for PRRSV entry and immune regulation [9,25,32]. This aligns with our findings that the mAb 28C10 exhibits a strong blocking effect on PRRSV. Previous research found that PK-15 cells became susceptible to ASFV after stably expressing Sn-CD163. It was observed that Sn and CD163 play a synergistic role in the ASFV infection process [19], which can explain why mAb 28C10 is able to block ASFV entry into PAMs. Other studies have found that the expression of Siglec-1 and CD163 is enhanced in ASFV-infected macrophages [65,66], indicating that Siglec-1 plays a role in the invasion of macrophages by ASFV. However, given the complex structure of ASFV, further studies are needed to clarify how Siglec-1 and CD163 receptors specifically facilitate ASFV entry into PAMs. Furthermore, we observed that ASFV replicated much less efficiently in PK-15^S1-CD163^ cells compared to PAMs (unpublished data), suggesting that additional receptors or mediators are likely involved in ASFV entry.

Another mAb 28G10B6 also exhibits a broad inhibition of PRRSV and ASFV, suggesting PRRSV and ASFV might use the same host factors facilitating their entry. Surprisingly, myosin heavy chain 9 (MYH9) was identified as the target protein of mAb 28G10B6 in PAMs (see Table S4). This identification was corroborated by another mAb against MYH9 [67], which also recognized a 260 kDa protein, confirming our finding (Figure 8d,e). MYH9, also known as non-muscle myosin heavy chain IIA (NMHC IIA), is a motor protein with a molecular weight of approximately 226 kDa [68]. MYH9 participates in numerous cellular processes, including cell migration, cytokinesis, and maintenance of cell shape [69]. MYH9 molecule has a long coiled-coil domain that allows two MYH9 molecules to interact, winding around each other to form a rod-shaped dimer which is essential for its role in building the larger, filamentous structures necessary for its contractile function [70]. This could potentially account for the presence of a prominent band exceeding 250 kDa in the input of Figure 8e, indicating a higher affinity of mAb 28G10B6 for the monomeric form of MYH9. MYH9 is ubiquitously expressed across various mammalian tissues and cell types [71]. MYH9 was highly positive on PAMs (Figure 8f). Immunofluorescence staining revealed that MYH9-positive cells are widely distributed in the nasal septum, nasopharynx, lungs, lymph nodes, and venous endothelial cells (see Figure S3). Furthermore, mAb 28G10B6 has demonstrated varying reactivity with CD14- and CD3-positive cells as analyzed by flow cytometry (Figure 1). MYH9 has been identified as a functional receptor for the infection of viruses, such as HSV-1 and Epstein–Barr virus [72,73]. Additionally, MYH9 is involved in the early infection of severe fever with thrombocytopenia syndrome virus (SFTSV) in vitro [74], and the activation of a Rho/NM II-dependent pathway facilitates the invasion of *Salmonella* or fusion of Sendai virus with host cells [75,76]. Furthermore, MYH9 has been identified as an important receptor for PRRSV infection, as it interacts with the viral GP5 protein and facilitates PRRSV entry in conjunction with the essential PRRSV receptor CD163 [10]. Consistent with this finding, our results demonstrated that mAb 28G10B6 had a strong blocking effect on the early stages of PRRSV infection. In addition, mAb 28G10B6 demonstrates a blocking effect on ASFV, indicating for the first time the involvement of MYH9 in ASFV entry into PAMs. However, the specific role of MYH9 in the process of ASFV entry in cells in this study has yet to be determined. One potential hypothesis is that the reorganization of MYH9 within cells could enhance the entry of ASFV. Upon infection with Herpes Simplex Virus-1 (HSV-1), MYH9, which is normally found in the cytoplasm, translocates to the cell surface [72,77]. Additionally, although PK15 is not susceptible to ASFV, it has the capability to express MYH9. Previous research has indicated that co-expression of CD163-Sn in PK15 cells can render them susceptible to ASFV [19], suggesting that MYH9 may collaborate with CD163 and Sn to facilitate ASFV replication. Consistent with this, we performed another PRRSV mAb blocking assay using different mAb combinations (see Figure S4), and we found that the mAb combination against Siglec1 and MYH9 exhibited a stronger blocking effect compared to the single mAbs, suggesting that MYH9 works together with Siglec1 as coreceptors for PRRSV. To further validate the role of MYH9, blebbistatin, a specific inhibitor of myosin II ATPase activity, can be introduced to assess its impact on ASFV replication in susceptible cells.

To date, numerous studies have developed antibodies against viral proteins to develop antiviral strategies. Previous research has found that the nanobody TAT-Nb1 can exert an antiviral effect against PRRSV by targeting the viral nucleocapsid protein [78]. Antibodies against PRRSV Nsp4 or Nsp9 have been shown to effectively inhibit viral replication [79,80]. However, as the virus continues to mutate and new viruses continue to emerge, antibodies targeting viral proteins may not provide reliable protection. In contrast, host receptors remain relatively conserved, making antiviral strategies targeting these receptors more likely to develop into broad-spectrum therapeutic drugs.

## 5. Conclusions

In summary, we have developed and characterized novel monoclonal antibodies specifically targeting PAMs, which effectively block the invasion of PRRSV and ASFV into these cells. We have also identified the PAM proteins targeted by these antibodies. Our findings shed light on the target proteins of these newly identified mAbs and highlight the critical role of these macrophage proteins in the early stages of PRRSV and ASFV infection. Future research will focus on elucidating their precise roles, potentially uncovering novel antiviral mechanisms.

## Figures and Tables

**Table 1:** Antibodies used for immunofluorescence staining or flow cytometry.

Primary Antibodies	Clone	Isotype	Dilution	Supplier
Mouse-anti-pig SWC3	4-22-15	IgG1	1:500	VMRD, Pullman, WA, USA
Mouse-anti-pig SWC3	4-22-15A	IgG2b	1:200	BD pharmingen, San Diego, CA, USA
Mouse-anti-pig CD3	PPT3	IgG1	1:200	Bio-Rad
Mouse-anti-pig CD3	BB23-8E6	IgG2b	1:500	Invitrogen
Mouse-anti-pig CD163	2A10/11	IgG1	1:200	Bio-Rad
Goat-anti-human CD163	Polyclonal	IgG	1:200	R&D Systems, Minneapolis, MN, USA
Mouse-anti-pig Sn	41D3	IgG1	1:50	[32]
Mouse-anti-human Sn	26B2	IgG2b	1:10	[33]
Mouse-anti-PRRSV N protein	13E2	IgG2a	1:100	[34]
Mouse-anti-ASFV (P72)	18BG3	IgG2a	1:100	Ingenasa, Madrid, Spain
Mouse-anti-ASFV (P72)	1BC11	IgG1	1:100	Ingenasa
Rabbit-anti-human ANXA1	Polyclonal	IgG	1:100	Invitrogen
Mouse-anti-human MYH9	5D9D2	IgG1	1:500	Proteintech, Sankt Leon-Rot, Germany

## Data Availability

Data is available upon request to the corresponding authors.

## Supplementary Materials

The following supporting information can be downloaded at: https://www.mdpi.com/article/10.3390/v17020167/s1, Figure S1: First round of selection of mAbs after virus blocking assay; Table S1: Analysis of mAbs (-FCS) reactivity with PAMs by IF staining; Figure S2: Second round of selected of mAbs analyzed in the blocking assay; Table S2: Summary of the most abundant proteins identified in the eluate of 26B8F5-I; Table S3: Summary of the most abundant proteins identified in the eluate of 28C10; Table S4: Summary of the most abundant proteins identified in the eluate of 28G10B6; Figure S3: Double Immunofluorescence staining against 28G10B6 (red) targeting MYH9 and CD163 (green) positive cells in different tissues; Figure S4: RRSV mAb blocking assay with different mAbs.
