# Supplementary material for: Monoclonal Antibodies Targeting Porcine Macrophages Are Able to Inhibit the Cell Entry of Macrophage-Tropic Viruses (PRRSV and ASFV)
Source: Viruses. 2025 Jan 24;17(2):167. doi: 10.3390/v17020167 (PMC11860747; doi:10.3390/v17020167)
Supplement: Supplementary file 1 [file viruses-17-00167-s001.zip › viruses-3428677-supplementary.pdf]

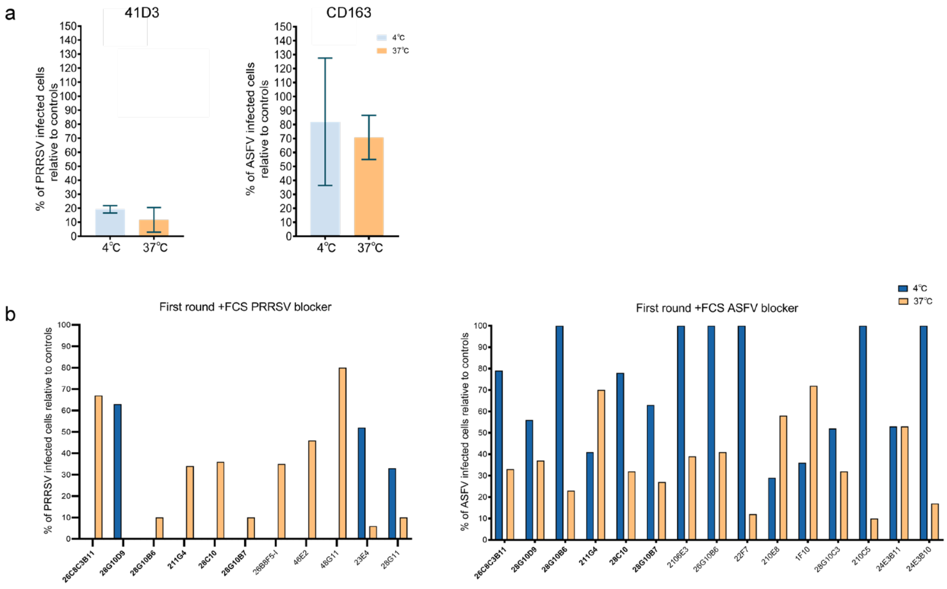

Figure S1. First round of selection of mAbs after virus blocking assay. (a) Positive control of virus mAb blocking assay. Anti-Siglec-1 mAb (41D3, IgG1) was used as a positive control in the PRRSV mAb blocking assay, while anti-CD163 mAb (2A10, IgG1) was used as a positive control in the ASFV mAb blocking assay. (b) After an initial screen, 11 mAbs out of 77 mAbs showed a blocking effect on PRRS (percentage of relative infection at 4°C: 0 to 52%; percentage of relative infection at 37°C: 6% to 80%), 15 mAbs out of 77 mAbs showed a blocking effect on ASFV (percentage of relative infection at 4°C:29% to 100%; percentage of relative infection at 37°C: 10% to 72%), and 6 mAbs (in bold) showed blocking effects on both PRRSV and ASFV.

Table S1 Analysis of mAbs (-FCS) reactivity with PAMs by IF staining.

mAb	IF staining (undiluted)	Concentration (ug/ml)
24E3B11	++	186
24E3B10	+++	367
210C5	++++	260
22F7/1F9	+++++	282
210E8/1H4	+++++	142
28G10C3	+++++	152
28G10B7	++++	123
28G10D9	+++++	201
28C10	+++++	275
28G10B6	+++++	367
46E2	+++	398
48G11	+++++	140
28G11	+++++	427
23E4	++	320
26B8F5-I	+++++	408

IF staining were performed on PAMs with new produced mAbs (-FCS). The PAMs were fixed and permeabilized with 100% MeOH before staining. The concentration of each mAb was measured by nanodrop.

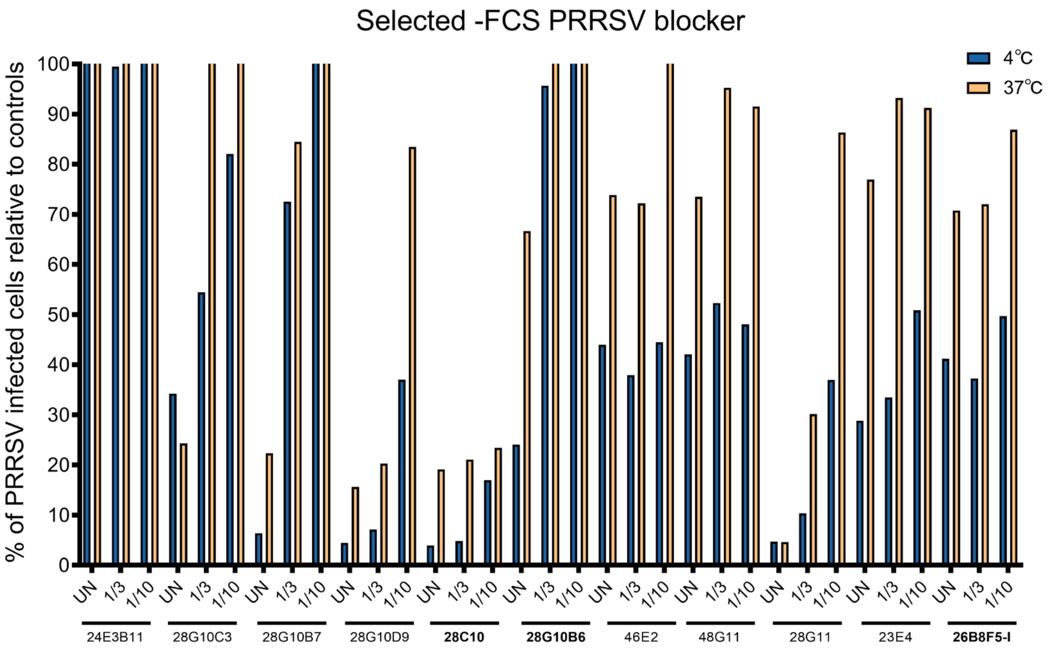

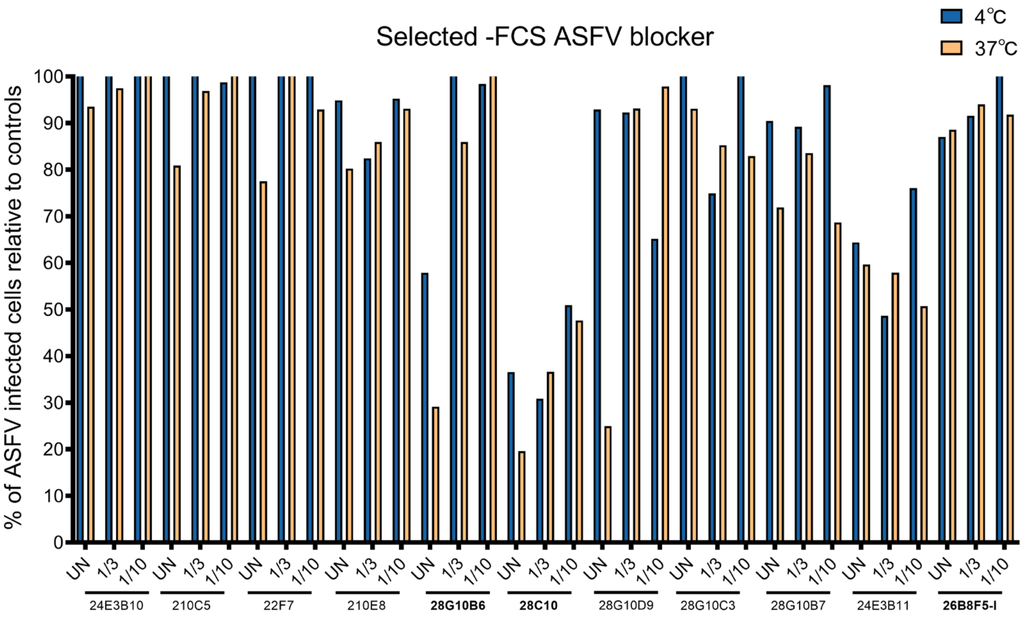

Figure S2 Second round of selected of mAbs analyzed in the blocking assay. a second-round screening of the mAbs without FCS was analyzed in the virus mAb blocking assay.

Table S2. Summary of the most abundant proteins identified in the eluate of 26B8F5-I

Gene name	Peptides	Sequence coverage [%]	Mol. Weight [kDa]	Score	MS/MS count
ANXA1	54	79.2	38.744	323.31	245
HSPA8	19	30.1	71.881	187.9	32
ALDOA	19	46.3	36.412	232.47	29
ACTG1	18	50.1	40.103	174.79	25
VIM	15	37.9	50.186	152.58	18
ACTR2	13	27.5	46.19	86.562	15

GAPDH	13	42.9	35.836	159.93	28
HSP90AB1	11	18.8	81.42	87.848	11
ANXA2	10	36.6	38.579	72.893	11
GIMAP4	9	25.9	36.688	58.807	8
EIF2S1	8	31.7	36.108	65.989	9
EIF3I	8	42.7	30.752	106.27	12
Histone H4	8	46.6	11.367	62.637	13
TALDO1	7	22.3	37.418	43.61	8
HNRNPA2B1	7	23.1	35.958	43.283	9
TUBB	6	18.8	47.635	54.988	9
CAPG	6	19	42.714	46.571	7
OAS1	6	16.9	40.246	41.04	7

Table S3 Summary of the most abundant proteins identified in the eluate of 28C10

Gene name	Peptides	Sequence coverage [%]	Mol. weight [kDa]	Score	MS/MS count
SIGLEC 1	51	31	185	323.31	291
Vimentin	35	60.9	53	323.31	76
ACTA1	7	18.1	41	54.639	10
Histone H2B	5	14.9	33	32.087	5
HSP90	4	7.1	83	24.465	4

Table S4 Summary of the most abundant proteins identified in the eluate of 28G10B6

Gene name	Peptides	Sequence coverage [%]	Mol. weight [kDa]	Score	MS/MS count
MYH9	256	69	222	323.31	1647
IQGAP1	100	55.7	195	323.31	226
MYO18A	104	51.4	233	323.31	191
SPTAN1	133	52	274.65	323.31	215
FLNA	46	23.6	283	24.465	53
MYO5A	21	12.1	214	149.25	23

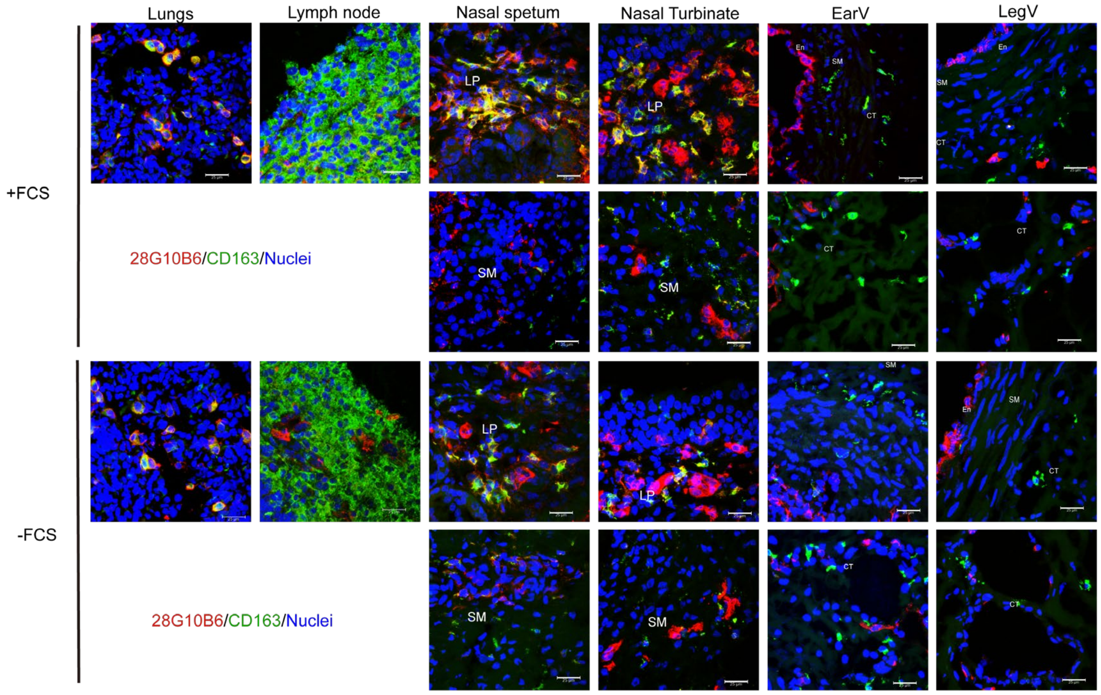

Figure S3. Double Immunofluorescence staining against 28G10B6 (red) targeting MYH9 and CD163 (green) positive cells in different tissues. Two different monoclonal antibody supernatants were used, the one produced with FCS (+FCS) and the one produced without FCS (-FCS). Nuclei were counterstained in blue (Hoechst 33342). Ear vein (EarV), Leg vein (LegV). Scale bar = 25μm.

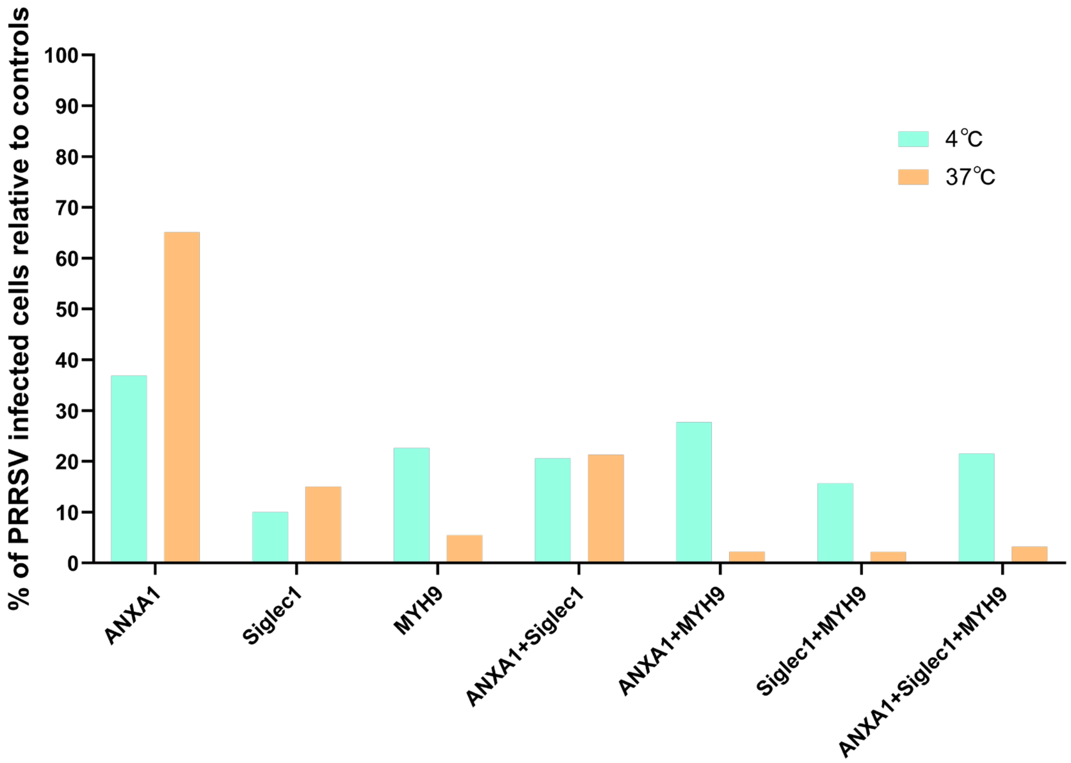

Figure S4. PRRSV mAb blocking assay with different mAbs. PRRSV mAb blocking assays were performed using different mAbs and different combinations.
